# Zebrafish as a Model for the Study of Live *in vivo* Processive Transport in Neurons

**DOI:** 10.3389/fcell.2019.00017

**Published:** 2019-02-19

**Authors:** Valérie Bercier, Marion Rosello, Filippo Del Bene, Céline Revenu

**Affiliations:** ^1^Institut Curie, PSL Research University, Inserm U934, CNRS UMR3215, Paris, France; ^2^Laboratory of Neurobiology, Center for Brain and Disease Research, Research Group Experimental Neurology, Department of Neurosciences, VIB-KU Leuven, Leuven, Belgium

**Keywords:** neuronal transport, zebrafish, myosin, kinesin, dynein, *in vivo*

## Abstract

Motor proteins are responsible for transport of vesicles and organelles within the cell cytoplasm. They interact with the actin cytoskeleton and with microtubules to ensure communication and supply throughout the cell. Much work has been done *in vitro* and *in silico* to unravel the key players, including the dynein motor complex, the kinesin and myosin superfamilies, and their interacting regulatory complexes, but there is a clear need for *in vivo* data as recent evidence suggests previous models might not recapitulate physiological conditions. The zebrafish embryo provides an excellent system to study these processes in intact animals due to the ease of genetic manipulation and the optical transparency allowing live imaging. We present here the advantages of the zebrafish embryo as a system to study live *in vivo* processive transport in neurons and provide technical recommendations for successful analysis.

## Introduction

Processive intracellular transport is essential for the distribution of organelles and cellular cargoes within the cell. In the case of neurons, such transport provides communication between different cell compartments and ensures supply to the growing synapse, clearance of detritus and serves as the support for intracellular signaling (Hirokawa et al., 2010). This process relies on the function of motor proteins and their interaction with the cell cytoskeleton, the three components of which play important roles in regulating transport. Indeed, neurofilaments provide structure and regulate axonal caliber, which influences transport metrics. Microtubules are responsible for axonal polarity, a consequence of the stereotyped orientation of their dynamic fast-growing ends, and act as the rails guiding motor proteins within the axon and dendrites. Finally, actin filaments form a structural network supporting the growth cone, pre- and postsynaptic regions, and play an important role in dendrites where they form the spines, essential for synaptic transmission.

Dynein and the kinesin superfamily are the unidirectional molecular motors responsible for transport on microtubules, both in dendrites and axon. In polarized axons, it is split according to the direction relative to the microtubule fast-growing end (+), with kinesins being responsible for ‘anterograde’ movement (toward the synapse) and dynein for ‘retrograde’ movement (toward the cell body). Their movement can in turn be categorized as ‘slow’ or ‘fast’ depending on their transport rate. Slow axonal transport is mainly used for delivery of cytoskeletal components and associated proteins, with kinetics in the range of 0.2–8 mm/day (Lasek et al., 1984). Fast axonal transport is used for organelles and vesicles, but also for mRNA granules (Maday et al., 2014), with kinetics in the range of 50–400 mm/day (Lasek et al., 1984). This type of transport, in terms of motor complex involved for different types of cargoes, motor adaptor complexes and transport metrics, has been widely studied and is well reviewed elsewhere (Maday et al., 2014).

Unconventional myosins, molecular motors of the actin cytoskeleton, are commonly associated with dynamic shaping of membranes, as well as organelle formation and transport, but their functions in neuronal transport is not well understood. Among this super-family, Myosin5a, 5b, 6 and 10 have been identified as processive transporters in neurons, and participate in local transport of intracellular cargoes over short-range distances. Processive myosins are likely part of a cooperative mechanism which is based on the coordination of actin and microtubule transporters (Wu et al., 2000). This is nicely illustrated with Myo5a binding directly to kinesins (Huang et al., 1999), suggesting that organelles transported in axons along microtubules may be transported by Myo5a in presynaptic terminals, which lack microtubules (Wu et al., 1998; Bridgman, 1999; Lalli et al., 2003; Nalavadi et al., 2012). Processive myosins are implicated in vesicle endocytosis, recycling and exocytosis, and hence participate in receptor transport and localization, regulating neuronal signaling and axonal pathfinding (Wu et al., 2002; Osterweil et al., 2005; Zhu et al., 2007; Correia et al., 2008; Nash et al., 2010; Lazo et al., 2013; Sui et al., 2015). Moreover, processive myosins take part in transport of mRNAs and RNPs in neurons, as demonstrated for Myo5a (Ohashi et al., 2002; Yoshimura et al., 2006; Balasanyan and Arnold, 2014; Calliari et al., 2014; Lindsay and Mccaffrey, 2014).

From recent evidence, it is apparent that *in vivo* axonal transport data do not recapitulate what has been observed *in vitro* (Gibbs et al., 2016; Klinman and Holzbaur, 2016; Knabbe et al., 2018), emphasizing the need for a more physiological context. With this in mind, excellent work has been published reporting *in vivo* axonal transport (reviewed in Sleigh et al., 2017) in models such as the *Drosophila* wing (Vagnoni and Bullock, 2017) and larvae (Vukoja et al., 2018), as well as the mouse brain (Knabbe et al., 2018) and sciatic nerve (Gibbs et al., 2016). All of these models have advantages and drawbacks: the mouse model is widely used and as a mammal, has a high genetic conservation of genes of interest but is not translucent and only allows access to axonal transport in a restricted area of the targeted cell population by way of surgery. The *Drosophila* is a model with a fantastic genetic manipulation toolbox, however, it is an invertebrate with reduced conservation to human compared to vertebrate models.

Over the last decades, zebrafish has emerged as a powerful vertebrate model to study the development of the nervous system *in vivo*. Adult zebrafish are small in size and produce a large number of offsprings, with a rapid external development. The embryonic zebrafish are translucent, and recent advances in genetic manipulation have made this model a great option to monitor neurodevelopment by high-resolution live imaging and at single-cell level. In addition, the zebrafish embryo is used extensively for modeling neurodegeneration (Bandmann and Burton, 2010; Kabashi et al., 2010; Santoriello and Zon, 2012; Babin et al., 2014; Patten et al., 2014; Fontana et al., 2018). Some processive motors have been associated with neurological disorders (Chen et al., 2013) and many studies have reported axonal transport defects in the context of neurodegenerative diseases (Chevalier-Larsen and Holzbaur, 2006; Goldstein, 2012; Liu et al., 2012; Millecamps and Julien, 2013), further outlining the interest of this model. In this article, we thus discuss the advantages of the zebrafish model in the study of live *in vivo* intracellular transport, with a particular focus on fast axonal transport.

## Advantages of the Zebrafish Model

### Relevance to Mammalian Models

The genome of *Danio rerio* is fully sequenced and presents at least one ortholog for 70% of human genes (Howe et al., 2013). In particular, kinesin, dynein and myosin molecular motors implicated in neuronal transports are extremely well conserved in eukaryotes and even more in vertebrates (Kim and Endow, 2000; Sittaramane and Chandrasekhar, 2008). These proteins have a higher conservation with the human ortholog in zebrafish compared to *D. melanogaster* for example. Zebrafish and drosophila dynein Dync1h1 show 91% and 72% identity (NCBI Blastp) with the human protein, respectively. Similarly, the processive Myo6 is 85% and 53% identical to the human one in zebrafish and drosophila, respectively. This high degree of conservation provides support for using zebrafish as a model system to investigate the functions of these molecular motors.

### Genetic Manipulations

Compared especially to the mouse, the ease of stable or transient genetic manipulations has positioned the zebrafish as an ideal vertebrate model for live *in vivo* imaging.

Transgenesis in zebrafish is routinely and efficiently performed to express fusion proteins, mutated proteins or the *gal4* transcription factor under a tissue-specific promoter thanks to the use of transposon elements. Ease of genetic manipulations in zebrafish has tremendously increased with the development of the CRISPR/Cas9 technology. The generation of knock-out mutants has become extremely powerful (Hwang et al., 2013) and using a Gal4/UAS-based restriction of Cas9 expression makes it possible to induce tissue-specific mutations and restrict the phenotype to a subset of cells (Di Donato et al., 2016). Recent advances based on the fusion of a mutated Cas9 (nickase) with an acetyl deaminase leading to the precise editing of a single nucleic acid (Komor et al., 2016) was also shown to work in zebrafish (Zhang et al., 2017). This technology makes it possible to target a specific protein domain in order to interfere with protein–protein interaction and opens the possibility of reproducing mutations associated with human diseases to elucidate the underlying pathological mechanism.

To recapitulate endogenous expression of a protein of interest, both in terms of pattern and level, bacterial artificial chromosome (BAC) transgenesis, where very large DNA sequence (up to 300 kb) can be inserted into the genome, is used in zebrafish (Lee et al., 2001; Suster et al., 2011). The CRISPR/Cas9 era has now opened the possibility of direct knock-in at a targeted locus. This strategy has been successful in zebrafish, based on the error-prone non-homologous end-joining DNA damage repair mechanism (Auer et al., 2014) and by short or long homology arm recombination (Hruscha et al., 2013; Hwang et al., 2013; Irion et al., 2014; Zhang et al., 2016). However, the efficiency of the latter technique is still low and locus-dependant. Its optimization will be an important technical advance in the field (Albadri et al., 2017), for example, to allow endogenous expression of a fusion protein of choice for visualization *in vivo*, without overexpression.

### Pharmacological Manipulations

Zebrafish embryos are amenable to pharmacological treatment by bath application, allowing for treatment of intact, live embryos with compounds known for the modulation of cytoskeletal dynamics, for instance, targeting microtubules: Colchicine (Roche et al., 1994), vinblastine (Keiter et al., 2016; Yao et al., 2017), vincristine (Mizgirev and Revskoy, 2010; Khan et al., 2012; Holloway et al., 2016), nocodazole (Plucińska et al., 2012; Jayachandran et al., 2016) and paclitaxel (Jayachandran et al., 2016). For the actin cytoskeleton: Cytochalasin D (Nukada et al., 2015; Artelt et al., 2018) and latrunculin A (Artelt et al., 2018), jasplakinolide (Artelt et al., 2018), phalloidin oleate (Dutta and Kumar Sinha, 2015), and the inhibitor of actin–myosin interaction BDM (Norden et al., 2009) have been used with success.

Finally, zebrafish embryos are well suited to high-throughput approaches that have made them an excellent tool in drug discovery by small molecule screening (Zon and Peterson, 2005; Mathias et al., 2012; Miscevic et al., 2012; Tamplin et al., 2012; MacRae and Peterson, 2015).

## Examples and Recommendations for the Analysis of *In Vivo* Transport in Zebrafish

To date, a few studies have taken advantage of the zebrafish model to perform *in vivo* axonal transport assays, generating tools to study the movement of mitochondria (Plucińska et al., 2012; Campbell et al., 2014; Paquet et al., 2014; Auer et al., 2015; Drerup et al., 2017), endosomes (Clark et al., 2011; Ponomareva et al., 2014, 2016), autophagosomes (He et al., 2009), lysosomes (Drerup and Nechiporuk, 2013), synaptophysin-containing vesicles (Auer et al., 2015) as well as motor proteins and components of their regulatory complexes (Drerup and Nechiporuk, 2013, 2016). The *in vivo* analysis of myosin-based transport is only starting in zebrafish neuronal development (Liu et al., 2013).

**FIGURE 1:**
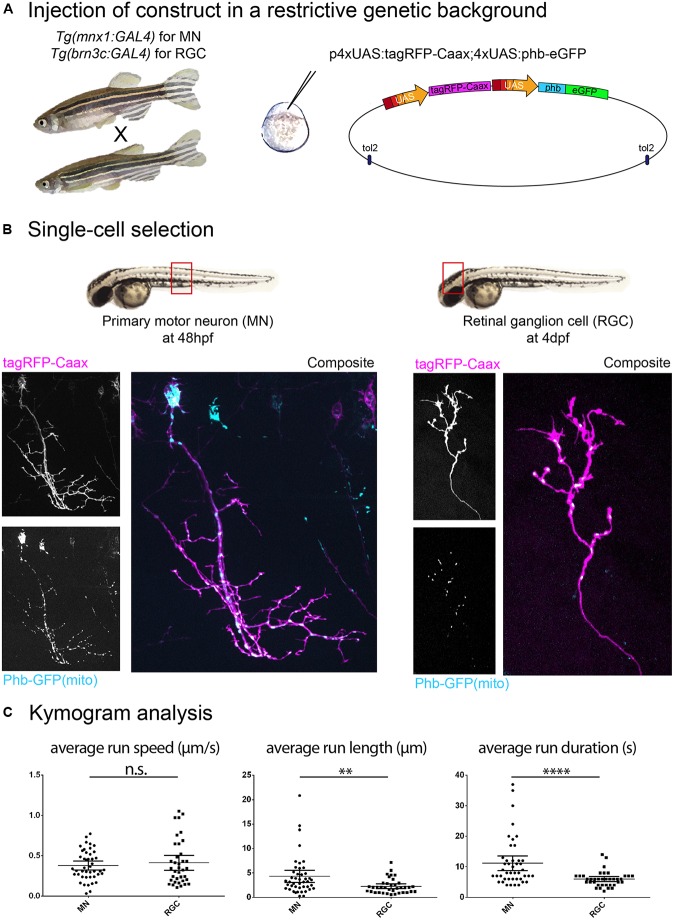
Construct expression and cell type selection. **(A)** Injection of DNA constructs coding for fusion proteins can be restricted to a single cell type by use of the Gal4/UAS system. **(B)** In this example, we injected a UAS construct labeling mitochondria (phb, prohibitin-GFP see schematics in **A**) combined with a membrane reporter (tagRFP-Caax). We obtained labeling of a single primary motor neuron (MN; in the *Tg(mnx1:gal4)* background) and a single retinal ganglion cell (RGC; in the *Tg(brn3c:gal4)* background), respectively, in the embryonic spinal cord (48 hpf) and in the larval optic tectum (4 dpf). **(C)** Time-lapse imaging of mitochondria (1 Hz for 10 min) was performed on these cell types, and transport dynamics were calculated from kymograms. Here, we show example of the disparity in transport metrics that can arise when comparing different cell types for a single cargo (MN *n* = 7 cells/44 anterograde runs; RGC *n* = 7 cells/37 anterograde runs). ^∗∗^*p* < 0.01, ^∗∗∗∗^*p* < 0.0001.

**FIGURE 2:**
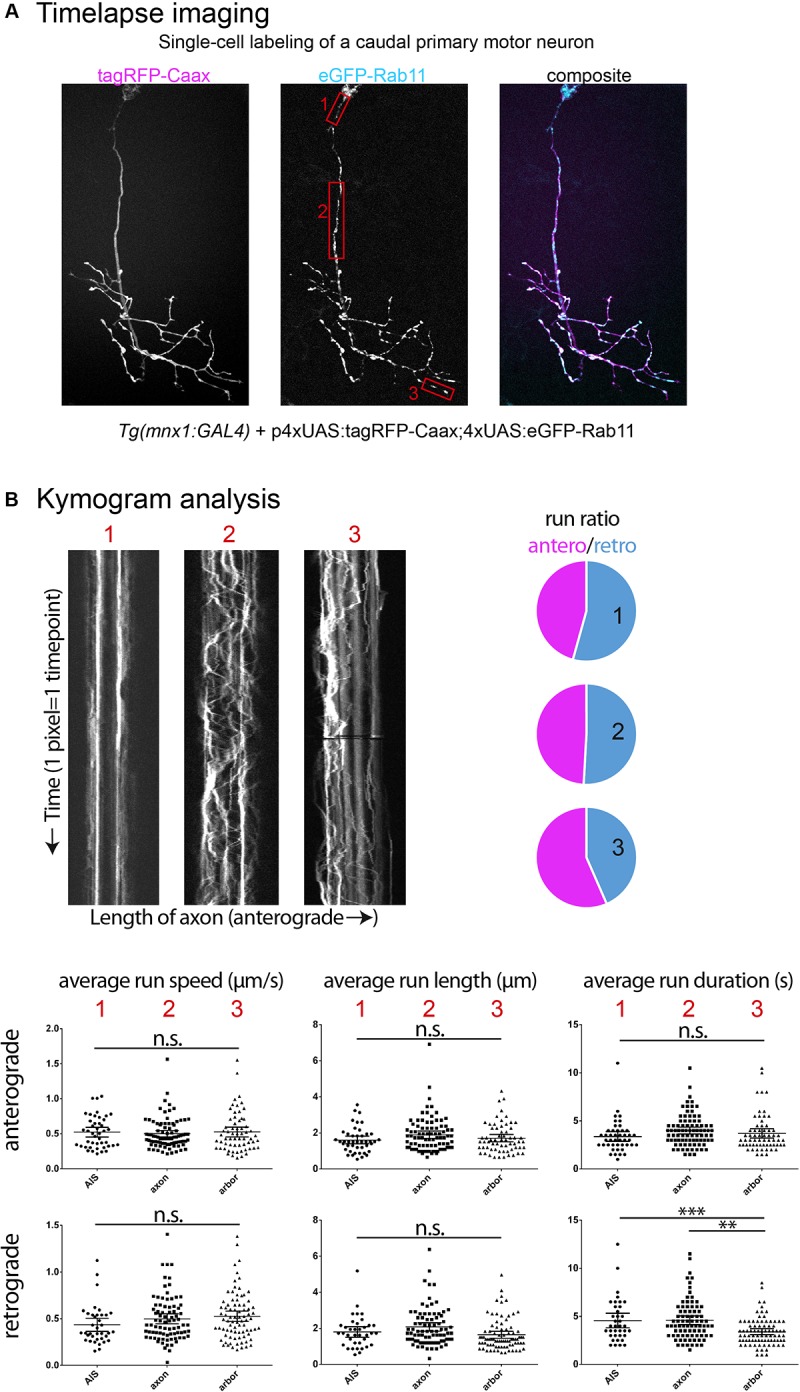
Examples of time-lapse analysis. **(A)** As described in Figure 1A, single cell labeling of primary motor neurons was obtained for recycling endosomes (Rab11a-eGFP), combined with a membrane reporter (tagRFP-Caax) to identify cell type. Red boxes: Three cell compartments were imaged (2 Hz for 5 min), 1-axon initial segment, 2-mid-axonal segment, 3-axonal arbor segment. **(B)** Kymograms were generated from the time-lapses acquired (Kymograph tool, ImageJ) and a variety of transport metrics can be calculated manually (compiled in Excel, statistics in Graphpad Prism6). In this example, significant differences between neuronal segments are detected for the transport direction ratio (anterograde/retrograde), and retrograde run duration (*n* = 3 cells; AIS *n* = 47/54 anterograde/retrograde runs; mid-axon *n* = 85/82 anterograde/retrograde runs; arbor *n* = 63/86 anterograde/retrograde runs). ^∗∗^*p* < 0.01, ^∗∗∗^*p* < 0.001.

Based on published evidence, it is plain to see that the metrics reported for the same cargo visualized *in vivo* in zebrafish display variation between cell types and developmental stages. Indeed, we have observed metrics for mitochondrial anterograde transport in primary motor neurons (MN; axon) and in retinal ganglion cells (RGC; arbor) and while we did not find differences in average run speed, average run length and duration were significantly different in these two cell types. Furthermore, the average run speed detected was approximately 0.4 μm/s (Figure 1C), which is consistent with reported data from Campbell et al. (2014) (peripheral sensory neuron arbors, approx. 0.4 μm/s) but inconsistent with data from Plucińska et al. (2012) (peripheral Rohon-Beard sensory neuron axons, approx. 1.2 μm/s ‘moving speed’ and 0.6 μm/s ‘average speed’) and from Drerup et al. (2017) (peripheral lateral line axon, approx. 1.0 μm/s). We also found discrepancies between cell types in the transport of recycling endosomes (labeled with Rab11a-GFP), where we observed an average speed of approx. 0.5 μm/s (Figure 2B), whereas Ponomareva et al. (2014) report an average speed of approx. 0.18 μm/s/0.03 μm/s (central/peripheral Rohon-Beard sensory neuron axon).

Based on the evidence above outlining the variability of these processes, we will highlight a few key points to take into consideration when designing experiments to characterize transport in zebrafish neurons.

### Regulation of Construct Expression and Imaging

Most approaches discussed here rely on the overexpression of fusion proteins, allowing *in vivo* detection of the bound fluorescent protein. This can be achieved injecting DNA constructs to obtain single-cell labeling of cargoes, as shown here (Figure 1A), or creating stable transgenic lines, where restriction of expression can be achieved by a combination of Gal4- and UAS-expressing lines. While this technique produces a bright signal well suited to time-lapse imaging, overexpression of protein can lead to deleterious effects by interfering with endogenous expression and triggering stress response mechanisms (Cheng and Lee, 2010). It is therefore essential to ensure that the construct does not lead to toxicity by monitoring cell morphology and embryonic development. The acquisition parameters in time-lapse microscopy are optimized to limit bleaching of fluorescent proteins and damage of the target cell, while still observing the target movement (for instance: high frequency sampling but reduced duration). In the case of the examples presented here, time-lapse imaging of labeled cargo in neurons was performed at 2 Hz for endosomes (5 min duration; Figure 2B) and at 1 Hz for mitochondria (10 min duration; Figure 1B,C) on a spinning disk confocal microscope.

### Single Cell Type

Axonal transport dynamics can be influenced by the varying expression of subunits composing the motor protein complexes or particular cargo adapters, as well as by the axon caliber, due to differences in microtubule density affecting engagement of motors (Yu et al., 2017) and due to activity- and myelination-dependent number of neurofilaments (de Waegh et al., 1992). It is therefore recommended to target one cell type (Figure 1B,C), and in the case of spinal cord neurons, to limit observation to a specific region as cell size can fluctuate along the trunk and tail owing to the rostro-caudal developmental wave.

### Single Cell Compartment

Different cell compartments have different cytoskeletal composition and regulatory mechanisms that can affect the composition and expression of motor proteins and their adaptor proteins, hence influencing the regulation of axonal transport (de Waegh et al., 1992; Yu et al., 2017; Gumy and Hoogenraad, 2018). As shown here (Figure 2), time-lapse imaging in different compartments of the same neuron for the same cargo can yield significant differences in some metrics of transport dynamics, but not others. The selection of a compartment most suited to the research hypothesis and consistency in the segment imaged across individual embryos and larva appears thus crucial, as well as the analysis of a variety of metrics such as run length and duration, average speed, pause frequency, average pause duration, switching behavior, area flux and transport rates of cargoes, both in the anterograde and retrograde directions.

## Perspectives for Future Research

### Alternative Labeling

Nanoparticles are inorganic semiconductors representing an attractive alternative for fluorescent labeling in live imaging applications because of their high spatial resolution and photostability. In addition, it is possible to tune their emission wavelength by varying their size and chemical composition. Because of this, and their broad absorption profile, it is possible to excite multiple colors at once, which is useful to reduce sample phototoxicity (Gao et al., 2005). In contrast to genetically encoded fluorescent protein tags, however, they need to be efficiently targeted to their biologically relevant endpoint. This has so far relied on surface modifications and solubilization strategies that led to very large particles better suited to high-sensitivity detection of low number of targets, such as single-molecule detection (Pinaud et al., 2006). Of note, this approach has allowed for real-time visualization of single-molecules in living cells (Dahan et al., 2003). Conjugation to biomolecules is, however, an interesting avenue to allow precise targeting and while still requiring the expression of a genetically encoded protein adaptor (Gao et al., 2005; Howarth et al., 2005), would provide the advantageous optical properties of nanoparticles over traditional fluorescent proteins.

### Microscopy Improvements

Advances in imaging technology in the last years have yielded many optimized systems applicable to the study of *in vivo* axonal transport in the zebrafish embryo. Indeed, a great example of this is the swept field confocal microscope, which permits higher frame-rate capture when compared with spinning disk confocal and allows the rapid acquisition of z-stack time-lapses or high speed imaging (upward of 1,000 fps) of movement in single-plane (Castellano-Muñoz et al., 2012). Other systems circumvent classic caveats to an *in vivo* approach, such as photodamage, single-plan and temporal restriction and low signal, for instance: 2-photon microscopy (Renninger and Orger, 2013), light-sheet microscopy (Huisken et al., 2004; Panier et al., 2013; Park et al., 2015; Tomer et al., 2015; Fu et al., 2016). In the context of *in vivo* imaging of axonal transport, these strategies could allow the tracking of cargo and motors with exquisite temporal resolution, while also permitting 3D tracking in a whole embryo over long periods of time; considerable advantages over *in vitro* and other *in vivo* models.

### Automated Detection and Analysis

The generation of kymograms as a 2D representation of time-lapse imaging is a common tool for the analysis of axonal transport, where the tracked target often moves on a single focal plane, in a linear trajectory. When analyzing movement in more complex environment, however, single particle tracking becomes a necessity, which renders manual analysis an arduous task. In the past years, many options have become available for automated detection and tracking, both commercially (Imaris, Metamorph, Igor Pro, etc.) and *via* open-source programs (MATLAB, ImageJ, etc.). Still, time-lapse videos obtained *in vivo* from intact animals are often noisier by nature than their cell culture counterpart, and since these samples are prone to photodamage, lead to undersampled data. This in turn impedes automatic detection and requires manual check of extracted metrics, while possibly omitting crucial information. Further advances in detection algorithms, based on *in vivo* data estimating how cargoes should behave, will surely be of benefit to researchers facing the tedious task of manual tracking.

## Conclusion

The zebrafish embryo has emerged as an excellent model to pursue the characterization of processive transport *in vivo* as it can meet the need for more inclusive models, where the contribution of neuronal activity, glia and the cell cytoskeleton are taken into account. We outlined here some advantages and technical hints to use the zebrafish model for this type of analysis. Considering the recent breakthroughs in genetic manipulations and imaging technologies, this vertebrate is gaining attention in the field of neurodegenerative disease modeling, where axonal transport deficits are common hallmarks. In addition, a new emerging model sharing the same subfamily as zebrafish, *Danionella translucida*, which remains transparent throughout its life, will further expand the possibilities of adult neuronal imaging *in vivo* (Schulze et al., 2018). It is thus only a matter of time before axonal transport assays in zebrafish embryos become widespread for the study of physiological and pathological conditions.

## Ethics Statement

As the EU directive 2010_63 explicitly states only “independently feeding larval forms” must be classified as animal experiments, therefore only zebrafish larvae past 120 h post fertilization should be subject to the regulations of European animal protection guidelines. For our experiments, we did not use larvae that have reached an “independent feeding” stage and therefore we did not have to submit an ethical approval to the competent local/national ethical/legal bodies.

## Data Availability Statement

The raw data supporting the conclusions of this manuscript will be made available by the authors, without undue reservation, to any qualified researcher.

## Author Contributions

All authors listed have made a substantial, direct and intellectual contribution to the work, and approved it for publication.

## Conflict of Interest Statement

The authors declare that the research was conducted in the absence of any commercial or financial relationships that could be construed as a potential conflict of interest.
